# Alpha-1 antitrypsin deficiency and risk of sleep apnea: a nationwide cohort study

**DOI:** 10.1007/s00405-025-09270-7

**Published:** 2025-03-14

**Authors:** Lucas Møller Larsen, Sine Voss Winther, Asbjørn Kørvel-Hanquist, Sarah C. W. Marott, Eskild M. Landt, Preben Homøe, Børge G. Nordestgaard, Morten Dahl

**Affiliations:** 1 Department of Clinical Biochemistry, Zealand University Hospital, 4600 Køge, Denmark; 2 Department of Otorhinolaryngology and Maxillofacial Surgery, Zealand University Hospital, Køge, Denmark; 3 Department of Clinical Biochemistry, Herlev-Gentofte University Hospital, Herlev, Denmark; 4 Department of Clinical Medicine, Faculty of Health Sciences, University of Copenhagen, Copenhagen, Denmark

**Keywords:** Genetics, Sleep disorder, Obstructive sleep apnea, Comorbidities, Epidemiology

## Abstract

**Objectives:**

α_1_-Antitrypsin deficiency is a disease characterized by increased neutrophil elastase activity leading to tissue getting less elastic and robust. It is known that if tissue in the pharynx becomes less elastic and robust, it could contribute to obstructive sleep apnea. This paper seeks to investigate whether patients with α_1_-antitrypsin deficiency have an increased risk of sleep apnea.

**Methods:**

We tested this hypothesis by doing a nationwide cohort study of 2702 individuals diagnosed with α_1_-antitrypsin deficiency compared with 26,750 individuals without α_1_-antitrypsin deficiency matched on sex, age, and municipality. All individuals were followed from birth and were censored at the time of outcome, emigration, death, or end of follow-up 31st of December 2018, whichever came first.

**Results:**

Individuals with α_1_-antitrypsin deficiency had a higher risk of sleep apnea with an adjusted hazard ratio of 1.81 (95% CI 1.36–2.40) compared to controls without α_1_-antitrypsin deficiency. Similarly, the risk of obstructive sleep apnea was nominally higher in individuals with α_1_-antitrypsin deficiency compared to controls without the disease (1.47, 95% CI 0.95–2.28). In stratified analysis, the risk of sleep apnea was higher in individuals without chronic obstructive pulmonary disease (2.33, 95% CI 1.54–3.51) (P for interaction < 0.05). The increased risk of SA was unaffected when the analysis was stratified by ischemic heart disease, ischemic cerebrovascular disease, type 2 diabetes, hypertension, and liver cirrhosis.

**Conclusion:**

Individuals with α_1_-antitrypsin deficiency have a higher risk of sleep apnea in the Danish population.

## Introduction

The function of the plasma protein α_1_-antitrypsin is to protect lung tissue, and other elastic tissues, from the activities of neutrophil elastase and other proteases [1]. Elastin is found in all tissues meant to be elastic and robust [2], especially in the lungs and to a lesser extent in the pharynx [3]. When level of elastase activity is heightened in individuals with α_1_-antitrypsin deficiency, elastin in the lungs will slowly be degraded, resulting in early-onset of chronic obstructive pulmonary disease (COPD) [4].

Obstructive sleep apnea (OSA) is a subtype of sleep apnea (SA) and is commonly caused by narrowing or collapse of the upper airways during sleep [5, 6]. Since α_1_-antitrypsin is a protease inhibitor and elastin is present in the pharynx, α_1_-antitrypsin deficiency could be causally related to OSA by degrading the upper airway tissues and increasing pharyngeal collapsibility [7, 8]. A previous study of 1386 adults showed that individuals with α_1_-antitrypsin deficiency genotypes had more symptoms and higher score on the STOP-BAG obstructive sleep apnea risk assessment questionnaire compared with controls in a propensity score matched analysis [8]. Individuals who have α_1_-antitrypsin deficiency could also have a greater susceptibility to sleep apnea due to hypoventilation or hypoxemia during sleep as they more often suffer from COPD [9].

We therefore hypothesized that individuals with α_1_-antitrypsin deficiency have an increased risk of overall SA and the subtype OSA. To test this hypothesis, we conducted a nationwide nested cohort study of 29,452 individuals from Denmark. The analysis was stratified for possible confounders like COPD, cardiovascular disease, and type 2 diabetes.

## Materials and methods

### Participants

For this nationwide nested cohort study, 2702 individuals diagnosed with α_1_-antitrypsin deficiency were identified in the Danish National Patient Registry, using the ICD10 code E88.0 (n = 1318), or genotyping/phenotyping information from the Danish Alpha-1 Deficiency Registry (ZZ, n = 1013; SZ, n = 168) and Copenhagen General Population Study (ZZ, n = 48; SZ, n = 155) [10]. Each of the case patients were matched with up to ten controls without α_1_-antitrypsin deficiency with regard to sex, age, and municipality, resulting in a control group of 26,750 participants. All individuals were followed from birth and censored at the time of outcome, emigration, death, or end of follow-up at 31st of December 2018, depending on what came first. The median follow-up time was 62 years.

Registry-based studies are exempt from approval from an ethics committee and informed consent requirements under Danish legislation. The investigation of individuals with α_1_-antitrypsin deficiency was approved by Herlev and Gentofte Hospital and Danish ethics committee (identification number H-KF-01-144/01) and was conducted according to the declaration of Helsinki.

### End-points/study outcomes

Sleep apnea (ICD10: G473*) was defined as having the primary or secondary diagnosis in the National Patient Registry. Obstructive sleep apnea (ICD10: G473.2) was similarly defined as having the primary or secondary diagnosis in the National Patient Registry. We also looked to include the ICD8 codes for “specific disorder of sleep” (ICD8: 306.4) and “disturbance of sleep” (ICD8: 780.6) but excluded these ICD codes from the diagnosis of SA due to few numbers of events (n < 5 events). The Danish National Patient Registry holds information about diagnoses from all Danish non-psychiatric hospitals since 1978, and from outpatient clinics, emergency departments and psychiatric departments since 1995 [11]. For the stratified analysis, we collected the following diagnoses from the Danish National Patient Registry: COPD (ICD8: 491–492, ICD10: J41–J44), ischemic heart disease (ICD8: 410–414, ICD10: I20–I25), ischemic cerebrovascular disease (ICD8: 432–435, ICD10: I63–I64, G45), diabetes mellitus type II (ICD8: 250, ICD10: E11, E13, E14), hypertension (ICD-8: 401–404; ICD-10: I10–13, I15) and liver cirrhosis (ICD8: 571.90–571.92, ICD10: K74). All the used information about the individuals, including diagnoses, were linked to the civil registration number, a unique social security number given at birth or immigration.

### Statistical methods

For the statistical analysis, Stata version 18.0 was used (StataCorp, College station, TX, USA). To analyze categorical data, we used Pearson’s χ^2^-test, and the Student’s t-test for continuous data. A two-tailed p-value of < 0.05 was considered statistically significant. The cumulative incidence curves of SA and OSA in individuals with and without α_1_-antitrypsin deficiency were modelled with Kaplan–Meier, with age as the underlying timescale. The Cox regression model was used to determine the risk of SA and OSA in individuals with α_1_-antitrypsin deficiency compared to age, sex, and municipality matched controls. The analyses were adjusted for sex and age and were also stratified by different comorbidities of α_1_-antitrypsin deficiency and SA to explore possible confounding. Stratified analysis was not done for OSA due to lower statistical power for this analysis.

## Results

We identified 2702 individuals with α_1_-antitrypsin deficiency and 26,750 controls matched on sex, age, and municipality. Table 1 shows no differences in distributions of age, sex, marital status, taxable income, and type of household between individuals with or without α_1_-antitrypsin deficiency. COPD and liver cirrhosis were more prevalent in those with α_1_-antitrypsin deficiency compared to controls, which is consistent with established knowledge about the possible comorbidities of α_1_-antitrypsin deficiency [4]. Individuals with α_1_-antitrypsin deficiency had also slightly lower prevalence of type 2 diabetes compared with controls.

**Table 1:** Characteristics of individuals with α_1_-antitrypsin deficiency

	Control group	α_1_-antitrypsin deficiency	P-value
Women/men	13,105/13,645	1323/1379	**0.98**
Age years	45.3 ± 20.3	45.5 ± 20.3	**0.76**
*Marital status*			**0.29**
Single	8086 (30.5)	796 (29.8)	
Married	13,909 (52.5)	1387 (51.8)	
Divorced	2875 (10.9)	318 (11.9)	
Widow/widower	1619 (6.1)	175 (6.5)	
*Taxable income (in quartiles)*			**0.21**
Low income	7767 (30.5)	835 (32.4)	
Low-Medium income	5511 (21.6)	536 (20.8)	
Medium–high income	5573 (21.9)	561 (21.8)	
High income	6623 (26.0)	644 (25.0)	
*Type of household*			**0.17**
Single male	2345 (9.9)	227 (9.5)	
Single female	3255 (13.8)	370 (15.5)	
Married couple	12,491 (52.8)	1233 (51.6)	
Other couples	2890 (12.2)	310 (13.0)	
Children (under 18) not living at home	< 5 (0,0)	0 (0,0)	
Other households consisting of multiple families	2666 (11.3)	252 (10.5)	
*Diagnoses*			
Sleep apnea	319 (1.2)	58 (2.2)	** < 0.001**
Obstructive sleep apnea	155 (0.6)	23 (0.9)	**0.08**
*Comorbidities*			
COPD	1817 (6.8)	1334 (49.4)	** < 0.001**
IHD	3158 (11.8)	347 (12.8)	**0.11**
ICVD	2013 (7.5)	196 (7.3)	**0.61**
Type-2 diabetes	1840 (6.9)	157 (5.8)	**< 0.05**
Hypertension	4858 (18.2)	514 (19.0)	**0.27**
Liver cirrhosis	149 (0.6)	136 (5.0)	** < 0.001**

Data is presented as n (%) or mean ± SD

*COPD* chronic obstructive pulmonary disease, *IHD* ischemic heart disease, *ICVD* ischemic cerebrovascular disease

### Risk of sleep apnea and obstructive sleep apnea

**Fig. 1:**
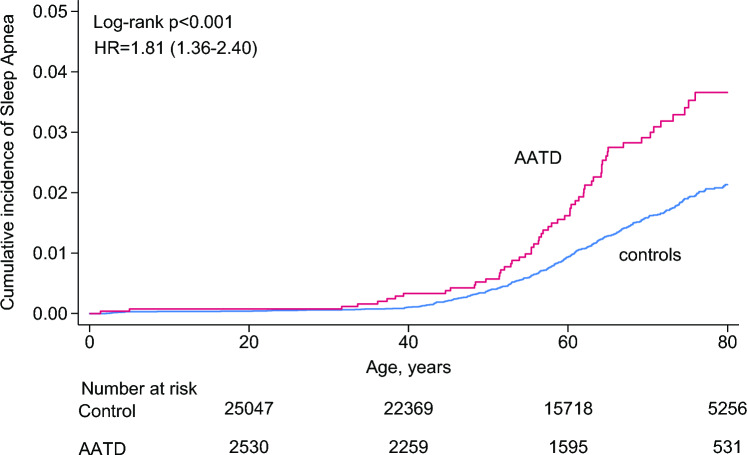
Cumulative incidence of sleep apnea in individuals with α_1_-antitrypsin deficiency compared to controls. The data are modelled with the Kaplan–Meier analysis. At 80 years and onwards, the graph flattens out as there are no further events and losses to follow-up. *AATD* α_1_-antitrypsin deficiency, *HR* hazard ratio

The survival analysis revealed that the cumulative incidence of developing SA was higher in individuals with α_1_-antitrypsin deficiency compared to controls (Fig. 1). When adjusting for sex and age, individuals with α_1_-antitrypsin deficiency had an increased risk of developing SA with a hazard ratio of 1.82 (95% CI 1.41–2.35) as compared to controls.

**Fig. 2:**
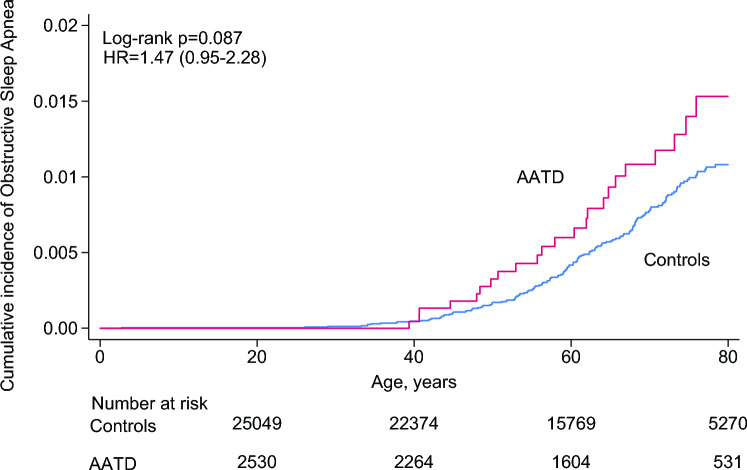
Cumulative incidence of obstructive sleep apnea in individuals with α_1_-antitrypsin deficiency compared to controls. The data are modelled with the Kaplan–Meier analysis. At 80 years and onwards, the graph flattens out as there are no further events and losses to follow-up. *AATD* α_1_-antitrypsin deficiency, HR hazard ratio

The same analytical approach was performed for OSA and showed similar, although non-significant results (Fig. 2). The hazard ratio for OSA in individuals with versus without α_1_-antitrypsin deficiency was 1.47 (95% CI 0.95–2.28) when adjusted for sex and age.

The cumulative incidence of SA at 80 years of age reached 3.5% in individuals with α_1_-antitrypsin deficiency compared with 2.0% in the control group. The cumulative incidence for OSA at 80 years of age was 1.4% in individuals with α_1_-antitrypsin deficiency compared with 1.0% in the control group.

### Stratified analysis

**Fig. 3:**
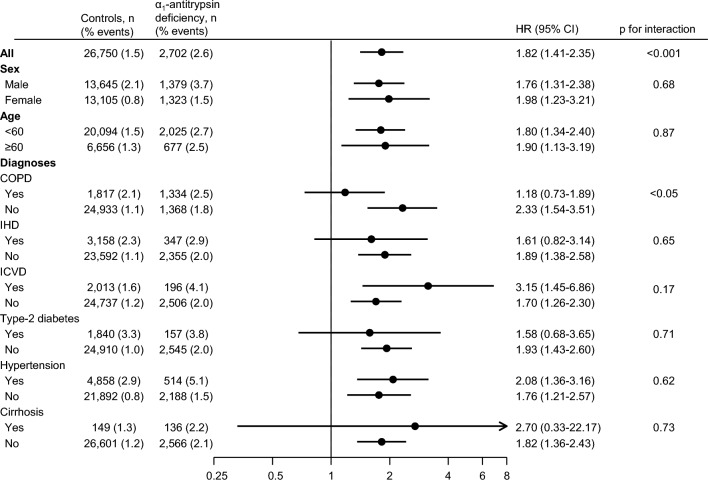
Stratified analysis of risk of sleep apnea in individuals with α_1_-antitrypsin deficiency compared to controls. Cox regression analyses adjusted for sex and age. *COPD* chronic obstructive pulmonary disease, *IHD* ischemic heart disease, *ICVD* ischemic cerebrovascular disease

We stratified for comorbidities of α_1_-antitrypsin deficiency and SA, including COPD, liver cirrhosis, ischemic heart disease [12], ischemic cerebrovascular disease [13], type-2 diabetes [14], and hypertension [15]. Additionally, we stratified for age and sex (Fig. 3).

The risk of SA in individuals with α_1_-antitrypsin deficiency differed between those having versus not having COPD (hazard ratio of 1.18 and 2.33 respectively, p < 0.05). Importantly, the risk of SA was highest among those without COPD suggesting that COPD did not drive the association observed between α_1_-antitrypsin deficiency and SA. The risk of SA in individuals with α_1_-antitrypsin deficiency did not differ significantly when stratified for the other comorbidities or risk factors in the analysis (p’s for interaction ≥ 0.15). Even though diabetes differed between the two study groups and OSA has formerly been linked to type-2 diabetes [14, 16], our results did not seem to be influenced by this possible confounder.

The risk of SA in individuals with versus without α_1_-antitrypsin deficiency was highest in individuals who had ischemic cerebrovascular disease (3.15, 95% CI 1.45–6.86), were without COPD (2.33, 95% CI 1.54–3.51), or had hypertension (2.08, 95% CI 1.36–3.16). The stratified analyses should be interpreted with care, due to reduced power in the subgroup analysis.

## Discussion

Our results show an increased risk of SA in individuals with α_1_-antitrypsin deficiency compared to controls with a hazard ratio of 1.82 (95% CI 1.41–2.35). Individuals with α_1_-antitrypsin deficiency had also a hazard ratio of 1.47 (95% CI 0.95–2.28) for developing OSA although this result did not reach statistical significance. The stratified analysis revealed that the results for SA could not be explained by common comorbidities of α_1_-antitrypsin deficiency or risk factors for SA such as COPD, liver cirrhosis, cardiocerebrovascular disease, or type-2 diabetes.

Within recent years it has become more evident, that α_1_-antitrypsin deficiency not only influences elastic tissue in the lungs, but also affect the skin [10] and the cardiovascular system [17]. Megenhardt et al. [8] conducted a cohort study where they investigated the relationship between α_1_-antitrypsin deficiency and OSA and found that individuals with α_1_-antitrypsin deficiency were more likely to have symptoms of OSA, e.g. snoring, tiredness, and observed apnea, and were thereby more likely to develop the diagnosis [8]. They speculated that the increased risk of OSA seen in individuals with α_1_-antitrypsin deficiency may only reflect the increased risk of COPD in individuals with α_1_-antitrypsin deficiency. We found that the risk of overall SA was higher among individuals without COPD (HR 2.33, 95% CI 1.54–3.51), thereby suggesting that there could be another underlying mechanism than COPD explaining the association. Further, the risk of SA did not differ by any other of the possible confounders analyzed. And when we did an additional sensitivity analysis on risk of OSA stratified by COPD, the highest risk was again observed in the group of individuals with α_1_-antitrypsin deficiency but without COPD (HR 2.75, 95% CI 1.58–4.78).

Megenhardt et al. included 118 severe cases (ZZ, SZ, null/S, null/Z genotypes) whereas our study included 2702 cases based on either genotyping information or diagnosis with the specific ICD10-code (E88.0), adding a substantial amount of data to Megenhardt et al.’s previous conclusion that there is a relationship between α_1_-antitrypsin deficiency and SA.

Megenhardt et al. did not specify the increased risk of having OSA, but the overall results are in accordance with our findings. Both studies have found a statistically significant relationship between α_1_-antitrypsin deficiency and features of SA. Because of our access to the Danish National Patient Registry, we were able to stratify for other comorbidities such as COPD, which Megenhardt et al. addressed as an important, missing variable in research on this issue, as carbon dioxide sensitivity, diaphragmatic mobility and frequent arousals at night in COPD contributes to the risk of OSA. Our results showed that the relationship between α_1_-antitrypsin deficiency and SA was strongest among those without COPD, suggesting that COPD could not explain the full association.

Nakanishi et al. conducted an analysis on the UK biobank, identifying 140 individuals with α_1_-antitrypsin deficiency and comorbidities [18]. Their findings revealed that individuals with α_1_-antitrypsin deficiency had a nominally hazard ratio of 1.3 (95% CI 0.3–5.1) for SA in an unadjusted analysis.

Because of lack of data and statistical power for the analysis of OSA, we did not describe the risk of OSA in more detail in a stratified analysis. The current definition of SA includes 13 different diagnoses including e.g. primary central sleep apnea (ICD10: G4731), which is an idiopathic disease [19], and Congenital Central Hypoventilation Syndrome (ICD10: G4735E) which is caused by a deficiency in the autonomic central control of ventilation and a global autonomic dysfunction [20], and thus is unlikely affected by α_1_-antitrypsin deficiency. When stratifying the SA diagnosis according to subtypes we found that 50% of individuals diagnosed with SA had sleep apnea without any subtype specified (G473), 48% had sleep apnea due to obstructive sleep apnea (G4732), and 2% had sleep apnea due to other subtypes with sleep apnea in sleep related respiratory disease not otherwise specified (G4730) and sleep related hypoventilation/hypoxemia due to medical illness (G4735) being the most prevalent. The higher hazard ratio for SA than for OSA observed in our study therefore seems primarily related to the diagnoses of “SA without any subtype specified” and OSA. Supporting that OSA may represent the largest fraction of cases in SA, a validation study of SA diagnoses in the Danish National Discharge Registry found that 80.3% of the patients with SA had an Apnea–Hypopnea Index of ≥ 5 (19.7% had no AHI recorded) and 80.3% of the patients were treated with CPAP, 18.2% received no CPAP, and 1.5% had unknown CPAP status [21]. Thus, the proportion of OSA in SA is likely about 80% in our cohort rather than 48% as observed in the stratified analysis. Even so, it should be noted that the positive predicted value (PPV) of SA is 80.3 percent, as opposed to the PPV for OSA of 93.8 percent [21].

Our diagnoses of SA and OSA probably reflect more severe cases, as former research finds that SA is an underdiagnosed disease in populations. It is estimated that 93% of women and 82% of men with moderate to severe SA have not been diagnosed [22], which will influence the risk estimates of both groups, possibly increasing it significantly from the presented 2% for the control group at 80 years and 3.5% for individuals with α_1_-antitrypsin deficiency at 80 years. An important limitation is that individuals with α1-antitrypsin deficiency presumably receive more medical attention, leading to the possibility of more diagnoses of SA and OSA. When we examined those who had clinical α1-antitrypsin deficiency with manifest COPD or liver disease, however, the risk estimates were not raised but were attenuated or remained (Fig. 3). When our analysis was limited to only those individuals who had α1-antitrypsin deficiency without knowledge of the condition in the Copenhagen General Population Study (n = 198), the results remained almost the same (HR for SA: 1.5, 95% CI 0.48–4.65) in line with similar sensitivity analyses of AATD related outcomes in our previous studies [17, 23]. Thus, we do not think that more medical attention for individuals with clinical overt α1-antitrypsin deficiency considerably influenced the findings in our current study.

A major strength in this study was our number of individuals with α_1_-antitrypsin deficiency (n = 2702), and the possibility to match each individual with up to 10 controls based on sex, age, and municipality, together with the long-term follow up. In this way, we minimized the influence from possible confounders. Furthermore, access to the complete Danish health registries reduced the risk of loss to follow-up. This study was done on the Danish population, which is primarily Caucasian. This makes the research less dependent on influence caused by ethnic differences, although it is uncertain if the association observed is applicable to other non-Caucasian populations.

## Conclusion

Our analysis suggests that the risk of SA is heightened for individuals with α_1_-antitrypsin deficiency in the Danish population. These results were independent from influence by possible confounders like age, sex, COPD, cardiocerebrovascular disease, and type 2 diabetes. The data also contributes new knowledge to the risk factors and possible pathophysiology underlying SA and OSA in the general population. Loss of elastin is presumably also a natural aging phenomenon in healthy individuals. Individuals with α_1_-antitrypsin deficiency could be a model for the natural aging process, a risk factor for OSA [24].

## Data Availability

Data in this study are available to other researchers upon reasonable request, application to Statistics Denmark or through a collaboration agreement with the authors. Summarized data and scripts for analyses are available according to Danish law.
